# Conversion Liver Resection for Hepatocellular Carcinoma with Portal Vein Tumor Thrombus after Single Tremelimumab Regular Interval Durvalumab Regimen as a Second-Line Therapy: A Case Report

**DOI:** 10.70352/scrj.cr.25-0721

**Published:** 2026-04-24

**Authors:** Diyar Akhmet, Akihiko Soyama, Ayaka Kinoshita, Hajime Matsushima, Hajime Imamura, Takashi Hamada, Ayaka Satoh, Kazushige Migita, Shun Nakamura, Baglan Askeyev, Masanori Fukushima, Tomohiko Adachi, Shinji Okano, Hisamitsu Miyaaki, Susumu Eguchi

**Affiliations:** 1 Department of Surgery, Division of Digestive Surgery and Transplantology, Nagasaki University Graduate School of Biomedical Sciences, Nagasaki, Nagasaki, Japan; 2 Department of Gastroenterology and Hepatology, Nagasaki University Graduate School of Biomedical Sciences, Nagasaki, Nagasaki, Japan; 3 Department of Pathology, Nagasaki University Graduate School of Biomedical Sciences, Nagasaki, Nagasaki, Japan

**Keywords:** hepatocellular carcinoma, single tremelimumab regular interval durvalumab regimen, sequential immunotherapy, portal vein tumor thrombosis

## Abstract

**INTRODUCTION:**

Hepatocellular carcinoma (HCC) is a leading cause of cancer-related mortality, with limited treatment options for advanced, unresectable cases. Although immune checkpoint inhibitors (ICI), particularly the combination of atezolizumab plus bevacizumab (Atezo+Bev), have improved outcomes, many patients ultimately experience disease progression. The single tremelimumab regular interval durvalumab (STRIDE) regimen has emerged as a promising alternative, yet evidence of its efficacy as a second-line therapy remains limited.

**CASE PRESENTATION:**

A 74-year-old man presented with recurrent HCC and liver cirrhosis complicated by portal vein tumor thrombosis (PVTT). Following 2 cycles of Atezo+Bev and 1 additional cycle of atezolizumab monotherapy, the tumor progressed rapidly. The patient was subsequently treated with dual ICIs (durvalumab and tremelimumab) under the STRIDE regimen, resulting in significant tumor shrinkage and normalization of serum tumor markers. After 4 cycles of STRIDE, conversion surgery was performed, including segment VI resection and portal vein branch thrombectomy. Histopathological analysis revealed >95% tumor necrosis and confirmed R0 resection.

**CONCLUSIONS:**

This case illustrates the importance of a multidisciplinary approach in the treatment of advanced HCC and shows that a successful response to the STRIDE regimen, even as a second-line therapy can enable curative conversion surgery while maintaining safety and tolerability in patients with limited hepatic reserve.

## INTRODUCTION

Hepatocellular carcinoma (HCC) is the most common form of primary liver cancer. It accounts for the majority of cases and, as of 2020, ranks as the sixth most frequently diagnosed malignancy and the third leading cause of cancer-related mortality worldwide.^1)^ Traditionally, the treatment of unresectable HCC was based on systemic therapy with tyrosine kinase inhibitors, such as sorafenib; however, the clinical efficacy of these agents remained limited.^2)^ The advent of immune checkpoint inhibitors (ICI) has significantly transformed therapeutic strategies. In the IMbrave150 trial, the combination of atezolizumab+bevacizumab (Atezo+Bev) demonstrated statistically significant improvements in both overall survival and progression-free survival compared with sorafenib, establishing this regimen as a new standard of care for first-line therapy.^3)^ Despite these advances, only about one-third of patients respond to Atezo+Bev, and most eventually experience disease progression, necessitating a change in therapy.^4)^ Subsequently, the HIMALAYA trial showed that the single tremelimumab regular interval durvalumab (STRIDE) regimen was also superior to sorafenib, achieving a 3-year survival rate of approximately 30% in patients with advanced HCC. This became the first approved dual checkpoint inhibitor regimen for HCC, combining anti-PD-L1 and anti-CTLA-4 antibodies.^5)^ In this context, conversion therapy has emerged as a promising strategy wherein systemic therapy is employed to achieve tumor downstaging, with the goal of enabling curative-intent surgical resection in patients initially diagnosed with unresectable HCC.^6)^ Early successes with this approach have been reported using Atezo+Bev as induction therapy—commonly referred to as the “ABC conversion” strategy. In patients achieving significant antitumor responses, R0 liver resection may become feasible, offering the potential for complete remission and discontinuation of systemic therapy.^4)^ In cases where Atezo+Bev proves ineffective, subsequent treatment is usually individualized, taking into account the patient’s clinical status and prior treatment tolerance.^7)^ This lack of standardized second-line strategies represents a major clinical challenge.

We report a case of advanced HCC with chronic hepatitis B and portal vein tumor thrombosis (PVTT), in which disease progression under first-line Atezo+Bev was followed by a marked partial response to second-line STRIDE therapy, enabling successful conversion surgery.

## CASE PRESENTATION

The patient was a 74-year-old male with a history of chronic hepatitis B, who previously underwent open hepatic resection for HCC. Histological examination confirmed HCC arising in the setting of liver cirrhosis. The postoperative course was uneventful, and the patient was followed regularly.

During follow-up, routine imaging revealed a locoregional recurrence in segment VI (S6) of the liver nearly 4 years after the initial hepatectomy. The small, localized recurrence without vascular invasion was located on the surface of the liver. Therefore, radio-frequency ablation was not indicated. The small lesion with preserved hepatic reserve supported the use of stereotactic body radiotherapy (SBRT), which was selected and administered (35 Gy in 5 fractions), resulting in disease stabilization.

**Fig. 1:**
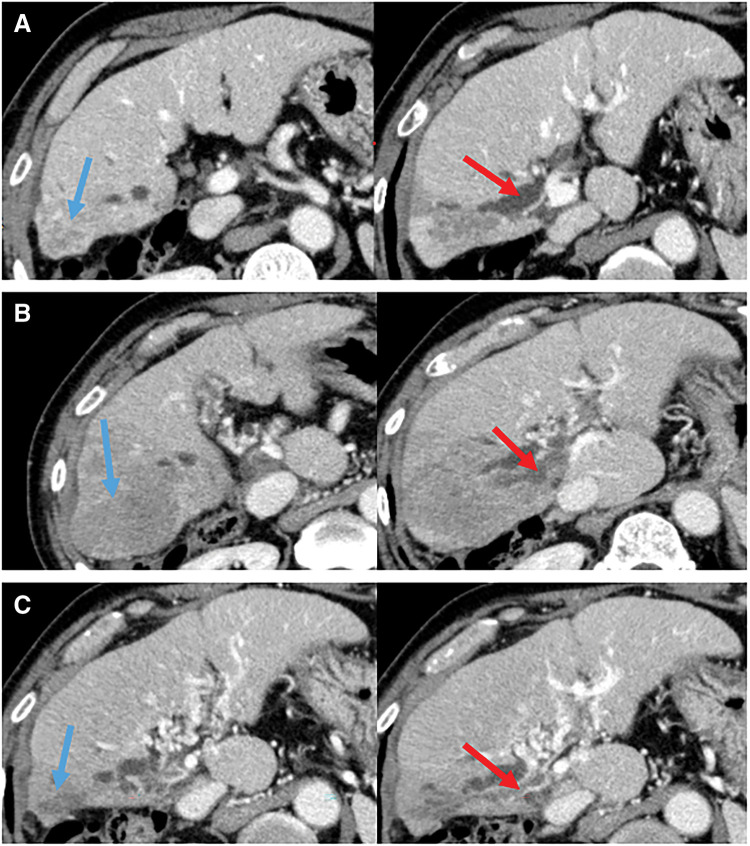
CT findings during treatment course. (**A**) Tumor in S6 of the liver at the time of recurrence diagnosis. (**B**) Progressive disease after 2 courses of Atezo+Bev, according to RECIST. (**C**) Partial response after 3 courses of durvalumab plus tremelimumab, according to RECIST criteria. Blue arrows indicate the lesion in liver S6. Red arrows indicate tumor thrombus in the right posterior branch of the portal vein. Atezo+Bev, atezolizumab plus bevacizumab; RECIST, Response Evaluation Criteria In Solid Tumors; S6, segment 6

Subsequently, 1 year later, imaging detected a new lesion within the previously irradiated area, suggestive of recurrent disease. Progressive tumor enlargement in S6(1.8 cm.) with invasion into the right posterior branch of the portal vein was later confirmed (**Fig. 1A**). The patient was diagnosed with recurrent HCC in a cirrhotic liver (Child–Pugh A5) with PVTT (Vp3), classified as cT3N0M0 and cStage III according to the General Rules for the Clinical and Pathological Study of Primary Liver Cancer, corresponding to Barcelona Clinic Liver Cancer (BCLC) stage C.^8)^

**Fig. 2:**
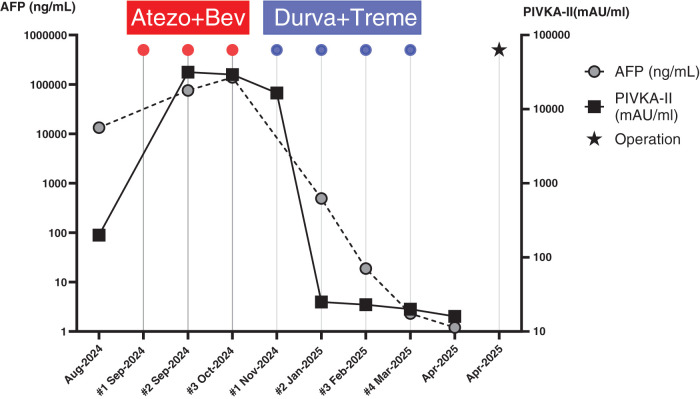
Changes in serum AFP and PIVKA-II levels during the course of immunotherapy. AFP, Alpha-fetoprotein; PIVKA-II, Protein Induced by Vitamin K Absence or Antagonist-II

Systemic therapy with Atezo+Bev (atezolizumab 1200 mg per body and bevacizumab 15 mg/kg) was initiated as first-line treatment, followed by a second cycle of atezolizumab monotherapy (1200 mg per body). However, follow-up imaging revealed further disease progression (PD) according to Response Evaluation Criteria In Solid Tumors (RECIST)^9)^: an increase in tumor size in S6 (4.9 cm.), persistence of PVTT (**Fig. 1B**), and a marked elevation in tumor markers (Alpha-Fetoprotein [AFP]–138514 ng/mL; Protein Induced by Vitamin K Absence or Antagonist-II (PIVKA-II)–29291 mAU/mL) (**Fig. 2**).

After progression on Atezo+Bev, a second course of hypofractionated radiotherapy targeting the portal vein was performed in (39 Gy in 13 fractions), followed by multidisciplinary discussion of further treatment options. Although lenvatinib is commonly used after Atezo+Bev failure, ICI-based sequencing was selected in this case due to preserved hepatic reserve (Child–Pugh A5) and concern for further liver function deterioration associated with progressive PVTT.

Given the need for prompt and effective disease control to preserve hepatic reserve and enable potential conversion surgery, and based on our institutional practice of avoiding concomitant lenvatinib during radiotherapy due to increased risk of hepatic deterioration and VEGF inhibition-related adverse events, combination immunotherapy with durvalumab and tremelimumab was selected as second-line therapy. The patient received a single priming dose of tremelimumab (300 mg) combined with durvalumab (1500 mg), after which durvalumab monotherapy was continued.

After 3 treatment cycles, partial response (PR according to RECIST) was observed: the lesion in S6 had decreased in size to 0.9 cm, the tumor thrombus in the right posterior branch of the portal vein showed regression (**Fig. 1C**), and serum tumor marker levels normalized (AFP–18.8 ng/mL; PIVKA-II–23 mAU/mL) (**Fig. 2**).

Taking into account the positive response against the background of STRIDE therapy, the technical feasibility of conversion hepatectomy has been multidisciplinary determined. Hepatic functional assessment showed adequate reserve for resection, with Liver-to-Liver plus Heart Uptake Ratio (LHL15) of 0.943 and Indocyanine Green Retention Rate (ICGR15) of 17.5%.

Following the favorable response, the patient received a fourth cycle of immunotherapy with durvalumab monotherapy (1500 mg per body) as part of the STRIDE regimen. Later evaluation demonstrated continued clinical improvement: the tumor in S6 further decreased in size to 0.5 cm., the portal vein tumor thrombus continued to regress, and tumor marker levels remained within the normal range (**Fig. 2**).

**Fig. 3:**
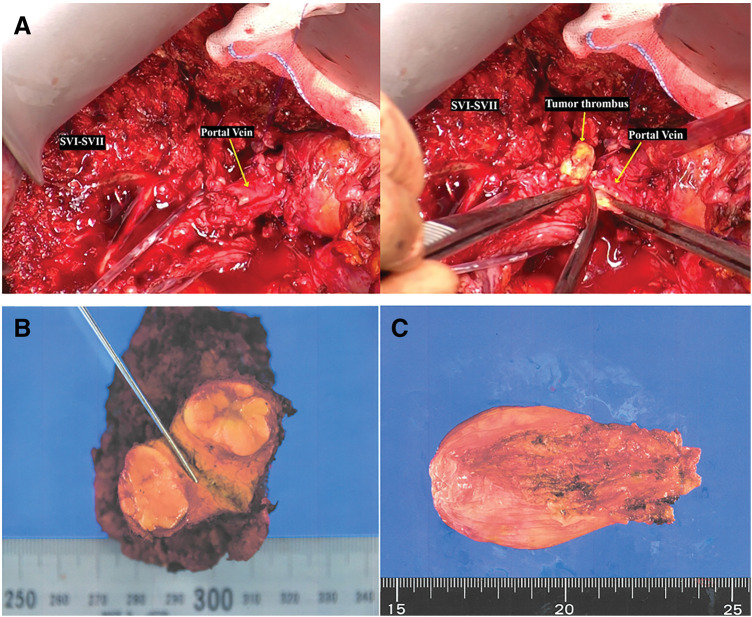
Intraoperative findings and resected specimens. (**A**) Right posterior section (S6–S7) and portal vein tumor thrombus intraoperatively; (**B**) Resected specimen showing the tumor in the right posterior section; (**C**) Removed portal vein tumor thrombus.

Approximately 5 months after initiation of second-line immunotherapy, the patient underwent open posterior sectionectomy of the right hepatic lobe with thrombectomy of the involved portal vein branch (**Fig. 3A**). Intraoperatively, dense adhesions related to prior hepatectomy and radiotherapy required extensive adhesiolysis for liver mobilization. In the presence of cavernous transformation of the portal vein, the surgical approach was individualized to preserve the right anterior portal vein branch. Prominent collateral circulation around the hepatic hilum complicated portal dissection and thrombectomy (**Fig. 3A**, **3C**), contributing to prolonged operative time and increased blood loss. The total operative time was 5 h and 41 min. Intraoperative blood loss was estimated at 1195 mL, and blood transfusion included 4 units of packed red blood cells, 4 units of fresh frozen plasma, and 10 units of platelet concentrate.

Histopathological examination revealed a small residual HCC (2 mm) (**Fig. 3B**) within the irradiated scar tissue, with marked treatment-induced necrosis of most tumor components. Additional immunohistochemical analysis demonstrated marked infiltration of CD4- and CD8-positive lymphocytes. PD-L1 immunohistochemistry was performed; however, evaluation of PD-L1 expression was limited due to the presence of only microscopic residual tumor. In addition, immunohistochemical staining for CTLA-4 showed positivity only in a small number of lymphocytes. The tumor showed a simple nodular growth pattern without capsule formation (fc−) or capsular invasion (fc-inf−), no vascular invasion (vp0, vv0), no bile duct invasion (b0), and no intrahepatic metastasis (im−). The pathological stage was pT1, and the background liver demonstrated cirrhosis (F4), according to the General Rules for the Clinical and Pathological Study of Primary Liver Cancer (7th Edition).

The early postoperative course was uneventful, and the patient underwent active rehabilitation. Follow-up monitoring is ongoing.

## DISCUSSION

This clinical case demonstrates the successful application of conversion therapy using the STRIDE regimen in a patient with initially unresectable HCC complicated by PVTT, following the failure of standard first-line immunotherapy. After tumor recurrence, the patient was treated with a first-line regimen of Atezo+Bev; however, disease progression was observed after only 2 cycles, indicating resistance to this combination.

Currently, there are no clearly established recommendations for second-line treatment following failure of ICI-based therapy, primarily due to the lack of well-designed randomized clinical trials. In the real-world clinical practice, treatment options often include tyrosine kinase inhibitors, ICI rechallenge, or locoregional therapies.^10)^ Clinical experience with tyrosine kinase inhibitors indicates that their antitumor activity is predominantly achieved through disease stabilization, whereas marked tumor regression is relatively uncommon, which substantially limits their potential in the context of conversion therapy.^11–13)^ Available data suggest that sequential switching from 1 ICI regimen to another is safe and can improve treatment outcomes in a substantial proportion of patients with HCC.^14)^ Several studies have demonstrated the efficacy of the STRIDE regimen as a second-line option after progression on Atezo+Bev. For example, in a retrospective study of 16 patients treated with Durva+Treme following failure of Atezo+Bev, disease control was achieved in 62.5% of cases, primarily due to tumor stabilization (SD according to RECIST).^15)^ In addition, individual case reports have shown that second-line treatment with Durva+Treme after ICI failure can lead to favorable outcomes, including PR.^7,16)^ In the present case, switching to Durva+Treme produced a pronounced clinical response, with partial tumor regression confirming sensitivity to ICI therapy enhanced by CTLA-4 inhibition.

The primary objective of conversion surgery was oncological consolidation with R0 resection after adequate tumor downstaging, particularly in the presence of PVTT, where durable disease control with continued systemic therapy is often limited and unpredictable. While a treatment-free (chemo-free) status was a potential benefit, surgery was prioritized during preserved liver function and confirmed resectability to avoid prolonged systemic immunotherapy of uncertain duration, cumulative toxicity, and loss of the surgical window.^17,18)^

Given the multimodal nature of treatment, hypofractionated radiotherapy was administered to the PVTT region before initiation of STRIDE. Considering prior reports that radiotherapy can enhance ICI efficacy, the PVTT regression with a reduction in tumor size observed after re-irradiation combined with durvalumab and tremelimumab likely reflects radio-immunotherapeutic synergy.^19,20)^

Preservation of hepatic functional reserve is a key prerequisite for the success of such treatment strategies, as it enables continuation of systemic therapy and is associated with improved overall survival.^21)^ Therapy with tyrosine kinase inhibitors may deteriorate liver function, whereas a sequential immunotherapy strategy, in the presence of preserved hepatic reserve, generally does not exert a pronounced negative impact.^22,23)^ The updated 4-year follow-up analysis of the HIMALAYA trial reported no new serious adverse events related to liver failure in the STRIDE group after the core observation period. The authors note that STRIDE preserves liver function and is not associated with delayed hepatotoxicity, supporting its favorable safety profile.^24)^ Real-world data further confirm that treatment with Durva+Treme maintains stable liver function parameters (modified Albumin–Bilirubin score and Child–Pugh class) during the early phases of therapy in patients with unresectable HCC, suggesting good tolerability even in patients with limited hepatic reserve.^25)^ In the presented case, liver function remained stable throughout the course of Durva+Treme treatment (shown in **Table 1**).

**Table 1:** Stable liver function parameters throughout the course of treatment in the STRIDE mode

	Oct. 2024 (PD)	Nov. 2024 (STRIDE#1)	Jan. 2025 (STRIDE#2)	Feb. 2025 (STRIDE#3)	Mar. 2025 (STRIDE#4)	Apr. 2025 (PE)
Albumin (g/dL)	3.9L	3.5L	3.9L	4.1	4.3	4.2
T-Bil. (mg/dL)	1.5	1.0	0.8	0.9	1.1	1.1
PT(%)	69L	72	85	83	79	80
INR	1.20H	1.19H	1.09H	1.09H	1.12H	1.13H
Ascites	Mild	Mild	Mild	Mild	Mild	Mild
HE	No	No	No	No	No	No
**Child–Pugh score**	6	6	6	6	6	6
**Child–Pugh grade**	A	A	A	A	A	A
**ALBI score**	−2.385	−2.161	−2.565	−2.702	−2.814	−2.729
**mALBI grade**	2b	2b	2a	1	1	1
AST(U/L)	37H	30	30	34H	41H	34H
ALT(U/L)	15	14	29	29	32	26
γ-GTP(U/L)	66H	66H	46	37	48	50

γ-GTP, gamma-glutamyl transpeptidase; ALBI score, albumin-bilirubin score; ALT, alanine aminotransferase; AST, aspartate aminotransferase; Dur+Trem, Durvalumab+Tremelimumab; H, high; HE, hepatic encephalopathy; INR, international normalized ratio; L, low; mALBI grade, modified ALBI grade; PD, progressive disease; PE, preoperative examination; PT, prothrombin time; STRIDE, single tremelimumab regular interval durvalumab; T-Bil., total bilirubin

A noteworthy aspect of this case is the presence of PVTT, which not only complicated surgical planning but also increased the clinical importance of achieving a response following the switch to the STRIDE regimen. Several reports have described significant regression of portal vein tumor thrombi during treatment with Atezo+Bev, enabling subsequent surgical intervention.^17,18)^ To date, there have been no published clinical cases describing conversion hepatectomy following treatment with the STRIDE regimen administered as a second-line therapy. Moreover, there are no reports of successful surgical resection after STRIDE treatment in patients with HCC complicated by PVTT, which indicates the need for further prospective studies.

The prolonged operative time and substantial intraoperative blood loss were primarily attributable to prior hepatectomy and repeated radiotherapy, which resulted in dense adhesions and difficult liver mobilization, as well as PVTT with cavernous transformation requiring complex portal dissection and thrombectomy. In addition, ICI therapy has been reported to induce inflammatory or fibrotic tissue changes that may increase tissue stiffness and adhesions, potentially further complicating surgical dissection, although direct evidence in liver resection remains limited.^26)^

The present case adds to the growing body of evidence on the efficacy of sequential immunotherapy and underscores that the STRIDE regimen can provide sufficient tumor control even in the presence of PVTT, thereby opening the possibility for radical surgical intervention in patients with initially unresectable disease.

## CONCLUSIONS

This clinical case supports the potential of the STRIDE regimen as an effective second-line therapy in advanced HCC following the failure of Atezo+Bev, resulting in marked tumor regression and normalization of tumor markers. The favorable response enabled curative (conversion) liver resection despite the presence of PVTT, confirming the feasibility of surgery after STRIDE treatment. Moreover, this case confirms the safety of the STRIDE regimen in patients with limited hepatic reserve, which further indicates its role in sequential immunotherapy strategies.

## DECLARATIONS

### Compliance with reporting guidelines

This case report has been prepared in accordance with the CARE guidelines for clinical case reporting. A completed CARE checklist is submitted alongside the manuscript.

### Availability of data and materials

The datasets generated and analyzed during the current study, including clinical details and images, were published with written informed consent from the patient. The data that support the findings of this study are not publicly available due to privacy reasons, but are available from the corresponding author upon reasonable request.
